# Character drawing style in cartoons on empathy induction: an eye-tracking and EEG study

**DOI:** 10.7717/peerj.3988

**Published:** 2017-11-08

**Authors:** Yong-il Lee, Yeojeong Choi, Jaeseung Jeong

**Affiliations:** 1 Department of Bio and Brain Engineering, Korea Advanced Institute of Science and Technology (KAIST), Daejeon, Republic of Korea; 2 HE Design Lab, LG Electronics, Seoul, Republic of Korea

**Keywords:** Empathy induction, Cartoon drawing style, Top-down process, Bottom-up process, Eye-tracking, Gamma oscillation

## Abstract

In its most basic form, empathy refers to the ability to understand another person’s feelings and emotions, representing an essential component of human social interaction. Owing to an increase in the use of mass media, which is used to distribute high levels of empathy-inducing content, media plays a key role in individual and social empathy induction. We investigated empathy induction in cartoons using eye movement, EEG and behavioral measures to explore whether empathy factors correlate with character drawing styles. Two different types of empathy-inducing cartoons that consisted of three stages and had the same story plot were used. One had an iconic style, while the other was realistic style. Fifty participants were divided into two groups corresponding to the individual cartoon drawing styles and were presented with only one type of drawing style. We found that there were no significant differences of empathy factors between iconic and realistic style. However, the Induced Empathy Score (IES) had a close relationship with subsequent attentional processing (total fixation length for gaze duration). Furthermore, iconic style suppressed the fronto-central area more than realistic style in the gamma power band. These results suggest that iconic cartoons have the advantage of abstraction during empathy induction, because the iconic cartoons induced the same level of empathy as realistic cartoons while using the same story plot (top-down process), even though lesser time and effort were required by the cartoon artist to draw them. This also means that the top-down process (story plot) is more important than the bottom-up process (drawing style) in empathy induction when viewing cartoons

## Introduction

Empathy is an essential function for human social activity, as it helps us to recognize relationships between others and ourselves by understanding others’ feelings, desires, ideas, and actions (Decety & Ickes, 2011; Yue, Pan & Huang, 2016; Zaki & Ochsner, 2012). Mass media, which has recently been used to distribute much of empathy-inducing content, plays a key role in individual and social empathy induction (Keen, 2007). From a psychological perspective, empathy is defined in terms of three individual components: self-other awareness, perspective-taking, and affective-sharing (Decety & Jackson, 2004). Self-other awareness involves two components: which are recognizing the distinction between the self and the other (awareness) and identifying oneself with a character (identification). Perspective-taking is the mental ability to take the subjective perspective of another person. Affective-sharing is the sharing of emotion between the self and the other, based on the perception action model, and it leads to shared representations.

From a cognitive neuroscientific perspective, empathy is a fundamental neural process that includes both the top-down and bottom-up aspects of information processing, as suggested by Jankowiak-Siuda, Rymarczyk & Grabowska (2011), and it could be indicated by the aesthetic experience of visual art (Freedberg & Gallese, 2007). The bottom-up process is based on the cognitive experience meditated by the sensory organs. In the case of a picture or a video, color, contrast, balance, symmetry, brightness, occlusion, the orientation of straight lines and curves, curvature, and convergence are bottom-up process components (Fuchs et al., 2011; Hamel et al., 2015; Leder et al., 2004). At the same time, the top-down process is performed by consideration of the understanding of the context of the story, intrinsic motivation, and surrounding atmosphere or individual differences: for instance, the viewers’ personal moods, cultural background, experience, education, training level, preferences, and personal interests in their own specific areas (Gilbert & Li, 2013; Graham et al., 2010). Many studies have revealed that frontal brain regions, including the anterior cingulate cortex (ACC), the anterior insula (AI), and inferior frontal gyrus (IFG), are related to empathy induction (Bernhardt & Singer, 2012; Decety, Michalska & Akitsuki, 2008; Fan et al., 2011; Lamm, Batson & Decety, 2007; Saarela et al., 2006). Based on these results, measurements of neural responses could be used for understanding top-down or bottom-up cognitive processes to empathic stimulus, through eye-tracking, functional magnetic resonance imaging (fMRI), magnetoencephalogram (MEG), electroencephalogram (EEG), and electromyogram (EMG) (Bölte et al., 2017; Balconi, Bortolotti & Gonzaga, 2011; Bloom, 2017; Cui et al., 2016; De Vignemont & Singer, 2006; Hein & Singer, 2010; Kang et al., 2014; Matsumoto et al., 2015; Suzuki et al., 2015; Vervoort et al., 2013).

In this light, the different iconic and realistic character drawing styles utilized in the present study seem to be highly related to bottom-up processes such as emotional contagion, while the empathy-inducing story used in the cartoons may be associated with top-down processes such as perspective-taking (Leiberg & Anders, 2006). Empathy is one of the important factors that elicits behavioral activities of cognitive responses when emotional scenes are presented (Keightley et al., 2010; Peled-Avron et al., 2016). This neural evidence provides us with not only a fundamental understanding of the neural correlates of empathy but also allows the development of more applied empathic content.

Based on the two main perspectives of empathy as described above, we aimed to investigate neurophysiological responses to empathy-inducing cartoons as well as the modulation of these responses by different styles of character designs to understand the influence of drawing style on the induction of empathy. Cartoons are a very historic and traditional media dating to ancient Egyptian wall paintings and continuing to 3D animation and virtual reality at present. Although cartoons are less realistic than photographs or films due to the uniqueness of their abstract style, they still serve as one of the most popular and informative forms of public media (Scott, 1993). Nevertheless, there is still a lack of quantitative data regarding whether cartoons are related to specific human emotions (Grubbs, 2016). To our knowledge, there is no research regarding how the different styles of cartoon modulate empathy.

We thus aimed to investigate the relationship between cartoons and empathy by answering the following questions: (1) Which of the top-down (story plot) and bottom-up (drawing style) elements in a cartoon contributes more to the induction of empathy? (2) How do the different drawing styles influence neurophysiological responses? We designed a cartoon with two different character drawing styles: iconic (closer to cartoon), and realistic (closer to photography). We used eye-tracking and EEG to assess the impact of the characteristics of the person that is observed in the cartoon on empathy.

## Method

### Participants

Fifty healthy Korean university students participated in this task. They had normal vision, were right-handed, and had no history of neurological disease (average age, 25.6 ± 2.6 years). The participants were divided into two groups corresponding to the individual cartoon drawing styles and were presented with only one type of drawing style. Participants in Group I watched only the iconic style cartoon and those in Group II watched only the realistic style cartoon. To confirm the homogeneity of our sample, the participants in each group were adjusted for age, gender, and education level. The ratio of male to female participants was approximately 2:1 (Group I is 17:8 and Group II is 16:9).

### General empathy scale for behavioral assessment

Prior to the main task, all the participants were asked to answer a preliminary questionnaire used to assess differences in the tendency to empathize using the relevant empathy scales. These empathy scales were defined as the General Empathy Scale (GES), which was adopted from the Multidimensional Emotional Empathy Scale (MEES) developed by Mayer and Caruso (Mayer, Caruso & Salovey, 1999; Neumann et al., 2015) and originate from Mehrabian’s research (Eisenberg, 2014; Mehrabian & Epstein, 1972). The task consists of completion of 30 questions and comprises six empathy subscales: “Empathic suffering”, “Positive sharing”, “Responsive crying”, “Emotional attention”, “Feeling for others”, and “Emotional contagion”. Scores for all subscales range from 1 to 5 points and are proportional to the degree of the corresponding feelings. In addition, overall empathy score of GES was obtained by averaging six empathy subscale scores. We performed two-sample *t*-tests to study differences in all individual and GES between the two groups. The averages and standard deviations of all empathy subscales for the two groups are summarized in Table 1. Two-sample *t*-test analyses revealed that there were no significant differences in any of the individual scales or GES between the two groups (*P*s > 0.05). These results show that there were no differences in the empathic ability between the two groups. All recruited participants were informed of the procedures used in this experiment and signed a written informed consent. They were paid approximately $30 for their participation. The study was approved by the Institutional Review Board at Korea Advanced Institute of Science and Technology (KAIST) (KH2016-43).

**Table 1:** Participants’ information and GES (general empathy scale).

	Group I (Iconic)	Group II (Realistic)	*P*-value
Participants number	25	25	N/A
Gender (Male:Female)	17:8	16:9	N/A
Age	20∼29	21∼29	
	(25.48 ± 2.65)	(25.72 ± 2.59)	0.747
Suffering	3.86 ± 0.55	3.90 ± 0.46	0.781
Positive sharing	3.89 ± 0.61	3.87 ± 0.59	0.925
Responsive crying	3.15 ± 0.84	3.24 ± 1.07	0.733
Emotional attention	3.63 ± 0.65	3.67 ± 0.51	0.810
Feel for others	3.40 ± 0.65	3.34 ± 0.55	0.710
Emotional contagion	3.61 ± 0.67	3.66 ± 0.34	0.721
Overall empathy score	3.59 ± 0.45	3.61 ± 0.33	0.861

### Experimental stimuli and paradigm

**Figure 1:**
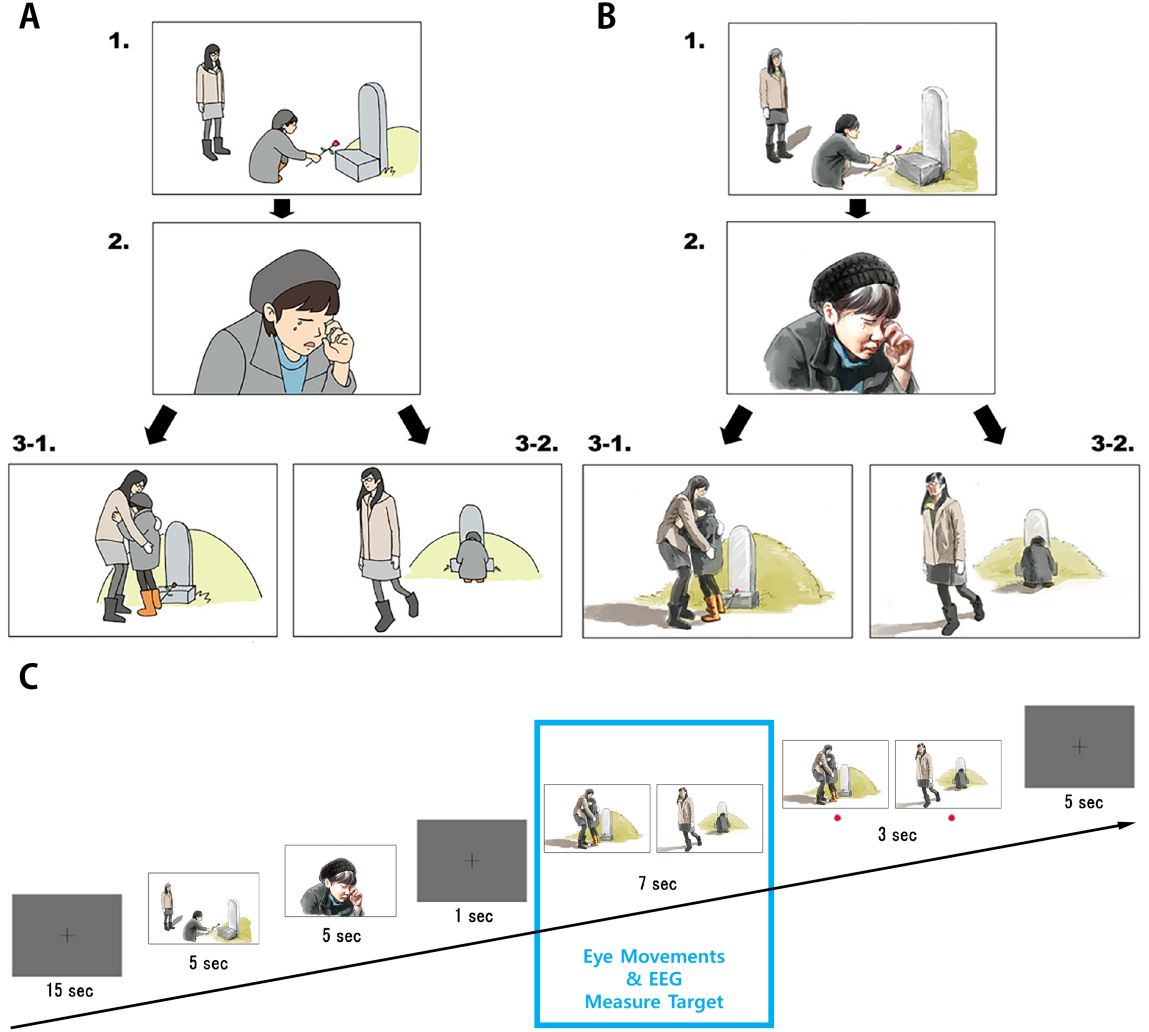
Schematic diagram of an empathy-inducing cartoon stimulus presented in two different drawing styles. (A) is iconic drawing style and (B) is realistic drawing style. (C) is the experiment procedure which has four steps except the black screen at the beginning and the end. Eye movements and EEG signals were recorded for 7 s prior to the presentation of the ending scene before choosing the ending scene.

Empathy-inducing scenarios consisting of a three-stage cartoon were designed to investigate changes in behavior and neurophysiological responses to the feeling of empathy. Ten kinds of cartoon scenarios were designed in advance. They were drawn from the third-person perspective and described sudden events that may happen in daily life and induce empathic emotions such as happiness, sorrow, anger, joy, excitement, pitifulness, surprise, remorse, amusement and embarrassment. Among them, four scenarios were eventually selected through an online pilot test, which selected for the scenarios resulting in more than 90% of the 65 participants (41 men and 24 women, with a mean age of 23.4 ± 1.2) responding with empathy induction. The final four scenarios were, a woman comforting a mourning girl in front of a grave (98.5%), a boy lifting a grandmother’s heavy load on her head (97.0%), a mother congratulating her son for winning a prize (95.4%) and a girl helping a woman splashed with water by a passing car (92.3%). The three-stage cartoons were based on a comic strip task used in a previous study conducted by Völlm et al. (2006). As shown in Figs. 1A and 1B, the first two stages of the cartoon explained the relevant background story, while the last stage consisted of one of two opposite conclusions of the story. These scenarios were presented using two different character drawing styles (iconic and realistic) to examine the resulting differences in the feeling of empathy.

### Main task procedure

The paradigm of main experimental task started with a black screen for 15 s as illustrated in Fig. 1C. This screen was sequentially followed by the first and second scenes with a story, which was shown for 5 s. Then, we showed a black screen for 1 s to control the baseline just before the third scene. Next, the two different ending scenes corresponding to the opposite conclusions were then presented for 7 s at the same time. Only the eye-tracking and EEG signals acquired during this session were analyzed. Finally, the participants were asked to select one of the two scenes (empathized or not empathized) that induced their empathic feelings more strongly by pressing a button when a red dot was displayed on the bottom of the screen for 3 s. This task was repeated four times, with the scenario changing every repeat. Participants who selected the not empathized scene more than two out of four times were excluded from the eye-tracking and EEG analysis.

### Induced empathy score for behavioral response

After the main task, we obtained the participants’ empathic responses to the scenario by surveying the relevant questionnaire adopted from the Decety and Jackson research, wherein the functional component of empathy is described in terms of three major factors: self-other awareness, perspective-taking, and affective-sharing (Decety & Jackson, 2004). Among them, we limited the meaning of self-other awareness to identification to make our task simple by referring to the study of Preston & De Waal (2002). We designed three questions corresponding to the three factors of empathy. The participants were asked to rate the following statements using a 7-point scale (1: strongly disagree to 7: strongly agree). The statements were (1) I felt as if I had become this character while viewing the cartoon (identification), (2) I came to think in the perspective of this cartoon character while viewing the cartoon (perspective-taking), and (3) I was sad when I saw this cartoon character crying (affective-sharing). The overall Induced Empathy Score (IES) was calculated by averaging the scores of the three statements. Finally, we asked the participants to write down the reason for empathizing with the cartoon. Answers that only consisted of a logical evaluation of the content or a self-report indicating a lack of empathy resulted in the exclusion of the participant from the study.

### Eye-tracking data recording and analysis

To extract the corresponding eye-tracking signals, we used a Tobii T120 table-mounted eye tracker (Tobii Technology, Stockholm, Sweden) integrated into a 17-inch thin film transistor monitor. Tobii Studio Software 1.5.12 was utilized for data acquisition, visualization, and analysis of eye movement data. The participants were seated comfortably at a distance of approximately 60 cm from the monitor. The stimulus was played at a resolution of 1,280 × 1,024. The eye-tracking rate was 60 Hz. Before starting the main experimental task, participants performed a calibration session to focus on nine sequentially appearing red dots presented in random placement on the screen.

In this study, the two ending scenes were defined as the areas of interest (AOIs) for which eye movements would be monitored. Two ending scenes were presented simultaneously on each one-half of the screen including enough blank backgrounds. Blank backgrounds were black and excluded from the AOI. We defined an eye fixation as gazing persistently at the same area. Usually the fixation area is set to approximately 40-pixel radius or less (Wieser et al., 2009), but we widened it further to 90-pixel radius to maximize our focus on comparison of AOI between the two ending scenes, rather than comparison among objects within each scene. Eight to nine fixations were classified as gaze durations of approximately 150 ms. Using the fixation definition, three parameters were measured for each AOI (Vervoort et al., 2013). (1) Time to first fixation and (2) first fixation length were indices of initial attentional processing and early orienting/allocation of attention. Subsequent attentional processing (attentional maintenance) was indexed by (3) total fixation length (gaze duration). ‘Time to first fixation’ was the time (latency) for initial attention orienting, which refers to the time taken from the onset of a scene stimulus to the first fixation on a specific AOI. When the ending scene was presented, gauging ‘Time to first fixation’ was stopped when the first fixation occurred in the central area of any of the two AOIs. ‘First fixation length’ was the duration of the first fixation that a participant made for each AOI of scenes before shifting to another AOI. ‘Total fixation length’ was the whole duration of the time for subsequent attentional processing (i.e., attentional maintenance) that a participant fixated on a particular AOI while the scene was presented. It was the total sum of each fixation length in the same AOI.

### EEG data recording and analysis

EEG signals were continuously recorded using a Neuroscan SynAmp I with a 32 QuickCap (Compumedics, Charlotte, NC, USA). This system contains 32 electrodes (passive AG/AgCl) mounted in an elastic cap and arranged according to the International 10–20 system. The right and left mastoid sites were used as reference electrodes. All impedance values were kept below 5 kΩ. The signal was digitized at a sampling rate of 1,000 Hz. Vertical and horizontal eye movements, including eye blinks, were monitored with extra two electrodes located approximately 1 cm above and below the left eye.

All following EEG analysis in this study were conducted using EEGLAB toolbox (Delorme & Makeig, 2004) on MATLAB (Mathworks Inc., Sherborn, MA, USA). We performed the pre-processing of the empathy-induced EEG signals as follows. First, the raw EEG signals were sampled at 1,000 Hz and down-sampled to 500 Hz by using the finite-impulse response band-pass filter from 1 to 100 Hz with a 60 Hz notch filter. Second, independent component analysis was performed to manually remove noise components from the EEG data, such as eye blinks, eye movements and facial muscle artifacts. In addition, two frontal polar EEG channels (Fp1/Fp2) were excluded from further EEG analysis to eliminate the effect of eye movements. Finally, EEG epochs were extracted from the continuous, artifact-corrected data, beginning 3,000 ms after the onset of the third ending scene. We used a baseline correction of the black screen from 500 ms to 1,000 ms, which was just prior to the third ending scene.

The preprocessed EEG epoch signals were obatined by means of a continuous wavelet transform based spectral analysis according to the procedures in the study of Tallon-Baudry et al. (1997). It has the advantage of extracting geometric mean of power at different scale ranges without the loss of frequency resolution (Avanzo et al., 2009). This method estimates the spectral power in the time frequency domain as defined in Eq. (1).

(1) \begin{eqnarray*}w(t,{f}_{0})=A\cdot {e}^{- \left( -{t}^{2}/2{\sigma }_{t}^{2} \right) }{e}^{2i\pi {f}_{o}t}, \text{with}~A={ \left( {\sigma }_{t}\sqrt{\pi } \right) }^{-1/2}\end{eqnarray*}

*A* is the normalization factor to normalize wavelets as the total energy value is 1. For this use, it was defined with *σ*_*f*_ = 1∕2*πσ*_*t*_. The constant ratio of this wavelet family (*f*_0_∕*σ*_*f*_) was set at 7 to increase the temporal resolution with frequency, whereas the frequency resolution had to decrease as a result. This indicates that the wavelet duration (2*σ*_*t*_) spanned approximately two periods long at the oscillatory activity (*f*_0_). The value of the wavelet family should be chosen in practice to be greater than 5 (Grossmann, Kronland-Martinet & Morlet, 1990; Scherbaum et al., 2011).

The calculated spectral powers were log-transformed and normalized by subtracting the mean power value in the baseline period of the black screen that was before the third ending scene. The time-varying gamma activities in this study were calculated by averaging the spectral power values within 30 to 50 Hz.

## Result

### Statistical analysis of IES

The averages and standard deviations of the IES subfactors for the two groups were as follows: (1) Identification (Group I: 3.48 ± 1.26; Group II: 3.52 ± 1.16), (2) Perspective-taking (Group I: 4.44 ± 1.19; Group II: 4.20 ± 1.26), (3) Affective-sharing (Group I: 4.84 ± 1.37; Group II: 4.44 ± 1.39), (4) Overall IES (Group I: 4.25 ± 1.09; Group II: 4.05 ± 1.14). Two-sample *t*-test analyses revealed that there were no significant differences in the three individual scores and overall empathy score between the two groups (*P*s > 0.05) (Table 2).

**Table 2:** Summary of IES (Induced Empathy Score) result.

		Group I (Iconic)	Group II (Realistic)	*P*-value
Induced empathy number (M:F)		23 (15:8)	24 (16:8)	N/A
Age		21∼2	21∼29	
		(25.78 ± 2.47)	(25.92 ± 2.45)	0.853
Identification	Total (*n* = 25)	3.48 ± 1.26	3.52 ± 1.16	0.908
	Only induced	3.57 ± 1.27	3.58 ± 1.14	0.959
Perspective-taking	Total (*n* = 25)	4.44 ± 1.19	4.20 ± 1.26	0.429
	Only induced	4.52 ± 1.20	4.29 ± 1.20	0.514
Affective-sharing	Total (*n* = 25)	4.84 ± 1.37	4.44 ± 1.39	0.311
	Only induced	4.91 ± 1.41	4.50 ± 1.38	0.317
Overall IES	Total (*n* = 25)	4.25 ± 1.09	4.05 ± 1.14	0.529
	Only induced	4.33 ± 1.10	4.13 ± 1.11	0.520

**Figure 2:**
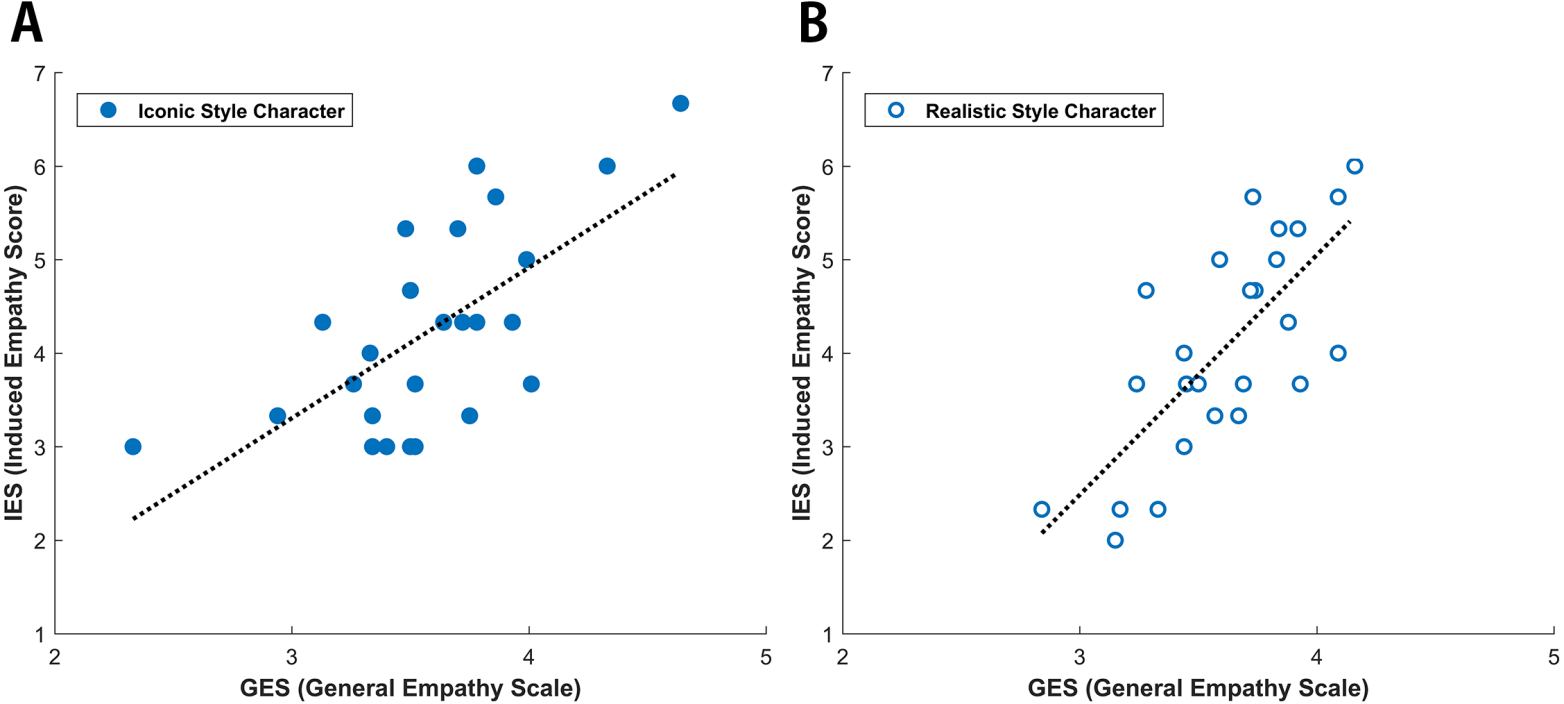
Linear regression analyses of IES (Induced Empathy Score) and GES (General Empathy Scale). The *x*-axis depicts the IES on a 5-point scale, while the *Y*-axis depicts GES on a 7-point scale. IES and GES were linearly correlated with one another in both iconic (*r* = 0.668, *p* = 0.000) and realistic (*r* = 0.740, *p* = 0.000) drawing style.

The number of participants who were induced with empathy was 23 in Group I (male: 15, female: 8) and 24 in Group II (male: 16, female: 8). They selected the empathized scene (left side of the third ending scene) more than three out of the four times. There were no significant differences ever after excluding the participants not induced with empathy (Group I: 2; Group II: 1). Furthermore, the gaps narrowed slightly between only the induced empathy participants of each group (*P*s > 0.05). In addition, IES was significantly correlated with GES under both the iconic (*r* = 0.668, *p* = 0.000) and realistic conditions (*r* = 0.740, *p* = 0.000), as shown in Fig. 2.

### Statistical analysis of eye movements

Due to the small number of participants not induced with empathy and the correlation between IES and GES, the analysis of eye movements was conducted only for participants induced with empathy (Group I: 23; Group II: 24). Like the IES result, two-sample *t*-test analyses revealed that there were no significant differences in the three eye movement parameters (time to first fixation, first fixation length, total fixation length) between the two groups (*P*s > 0.05) (Table 3). These results were consistent in the two AOIs, for both the empathized scene (left side of the third ending scene) and the not empathized scene (right side of the third ending scene).

**Table 3:** Summary of eye movement result.

Parameter	Scene	Group I (*n* = 23)	Group II (*n* = 24)	*P*-value
Time to first fixation (second)	Empathized (Left)	0.30 ± 0.12	0.28 ± 0.14	0.688
	Not empathized (Right)	0.28 ± 0.10	0.28 ± 0.14	0.985
First fixation length (second)	Empathized (Left)	0.63 ± 0.31	0.65 ± 0.24	0.787
	Not empathized (Right)	0.69 ± 0.29	0.65 ± 0.31	0.665
Total fixation length (second)	Empathized (Left)	3.98 ± 1.14	4.12 ± 0.56	0.596
	Not empathized (Right)	1.13 ± 0.32	1.09 ± 0.08	0.543
	Out of AOIs	1.89 ± 0.95	1.79 ± 0.55	0.667

**Figure 3:**
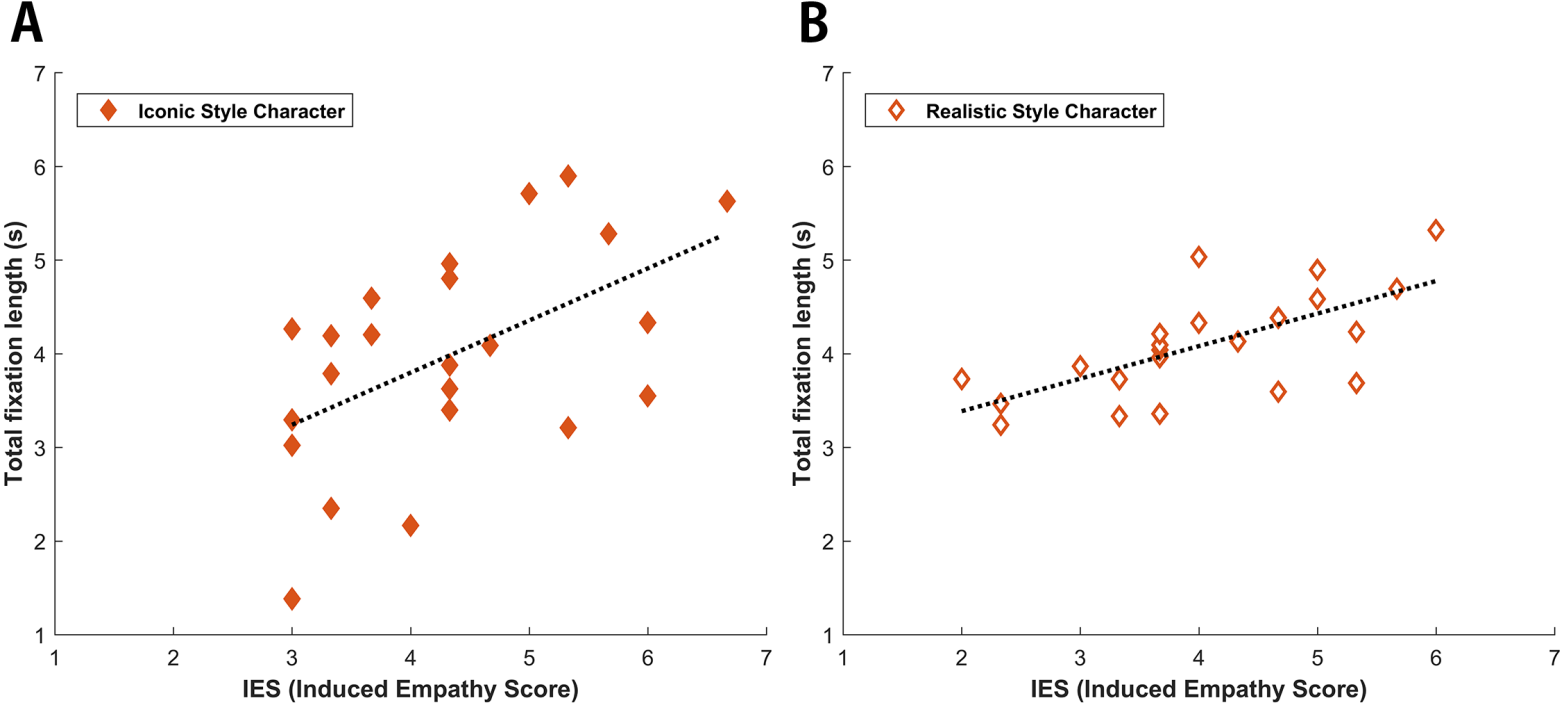
Linear regression analyses of IES and total fixation length. The *x*-axis depicts the IES on a 7-point scale, while the *Y*-axis depicts the total fixation length, up to a maximum of 7 s. IES and total fixation length were linearly correlated with one another in both iconic (*r* = 0.534, *p* = 0.008) and realistic (*r* = 0.687, *p* = 0.000) drawing style.

One the other hand, linear regression analyses indicated that there was a close relationship between the IES and the total fixation length of the empathized scene, as shown in the left of Fig. 3. These measurements were linearly correlated under both the iconic (*r* = 0.534, *p* = 0.008) and realistic conditions (*r* = 0.687, *p* = 0.000), as shown in Fig. 3. This indicated that higher degrees of empathy were associated with subsequent attentional processing (gaze duration). There were no relationships between IES and other eye movement parameters of initial attention orienting (time to first fixation: iconic (*r* = 0.138, *p* = 0.530), realistic (*r* = 0.027, *p* = 0.900); first fixation length: iconic (*r* = 0.082, *p* = 0.711), realistic (*r* =  − 0.198, *p* = 0.353)). Additionally, total fixation length of the not empathized scene (right) was significantly shorter than the total fixation length of non- AOI regions under both the iconic (*t*(22) = 3.608, *p* = 0.001) and realistic (*t*(23) = 6.155, *p* = 0.000) drawing styles.

### Spectral analysis and topography of EEG

**Figure 4:**
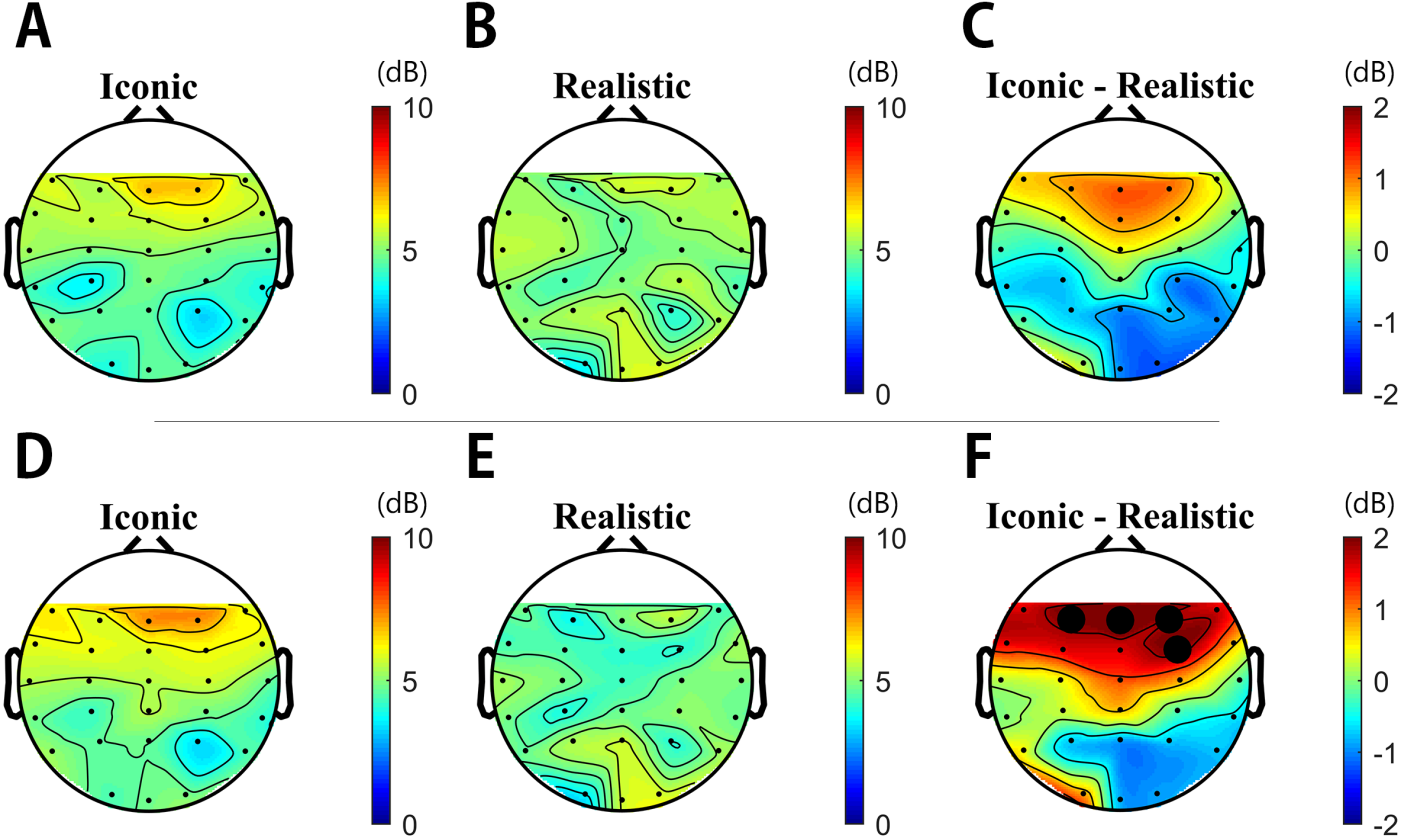
Topographies obtained via spectral analysis based on continuous wavelet transformation (CWT). (A–C) from 0 ms to 3,000 ms and (D–F) from 1,000 ms to 1,600 ms. Electrode power for F3, F4, Fz, and FC4 was significantly higher for the iconic condition than the realistic condition during the same time interval (*P*s < 0.05).

To demonstrate the effect of drawing style on EEG activity, we first examined the differences in spectral power between the iconic and realistic drawing styles for the 3,000 ms duration. Except for the activity in gamma band, we did not find any statistical differences in other frequency bands. Paired *t*-test analyses with multiple comparison by means of false discovery rate examined the statistical differences in gamma activities between the two different drawing styles. It revealed that the effect of drawing styles strongly modulated the fronto-central regions rather than other brain regions. Specifically, Fig. 4 illustrated the overall spatial and temporal characteristics of empathy-induced frontal gamma activities.

**Figure 5:**
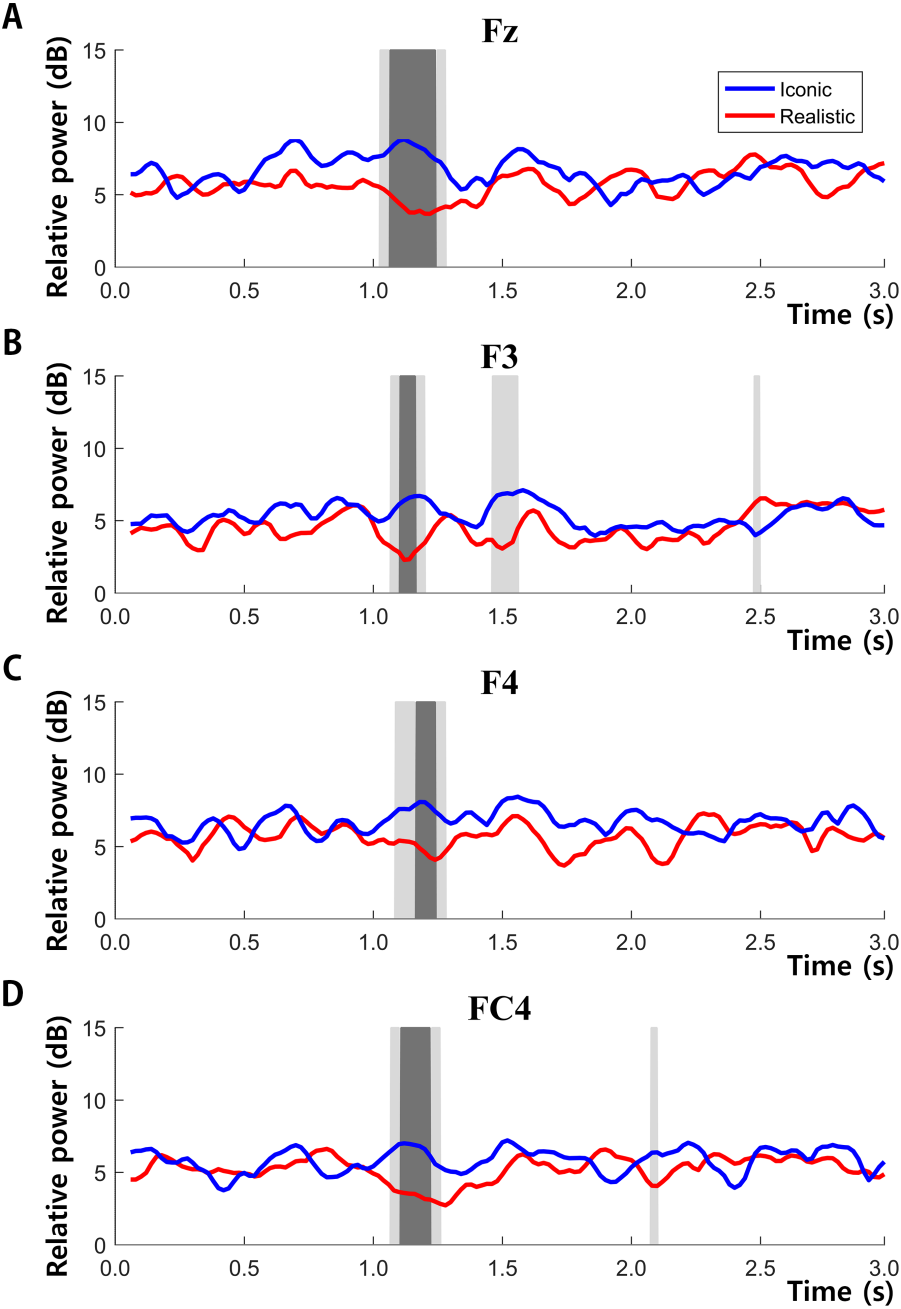
Time-series graphs of power and significant difference periods for the F3 (A), F4 (B), Fz (C), and FC4 (D) electrode. The *p* value of the dark gray block is under 0.01, while that of the light gray block is under 0.05.

Figures 4A–4C (within 0 ms to 3,000 ms) and 4D–4F (within 1,000 ms to 1,600 ms) illustrates the topographies of gamma activity between the two different drawing styles respectively. In all durations, gamma power was highly enhanced over the fronto-central area (F3/Fz/F4/FC4) during the iconic condition compared to those in the realistic condition. In Fig. 5, frontal gamma powers of four electrodes were significantly higher in the iconic condition than in the realistic condition briefly from 1,000 ms to 1,600 ms (*P*s < 0.05 interval is filled light gray, *P*s < 0.01 interval is filled dark gray). Figures 4D–4F topographies shows the spatial distribution of differences in gamma power between the two drawing styles with the assigned time segment (from 1,000 ms to 1,600 ms): gamma powers in the F3, F4, Fz, and FC4 channel were significantly higher in the iconic condition than in the realistic condition at the same time interval (*P*s < 0.05) (F3 (*t*(46) = 2.230, *p* = 0.036), Fz (*t*(46) = 2.719, *p* = 0.012), F4 (*t*(46) = 2.181, *p* = 0.039), FC4 (*t*(46) = 2.401, *p* = 0.024)). Consequently, these analyses revealed that different drawing styles mainly elicited the fronto-central gamma activities during early mid-term of stimulus presentation.

## Discussion

In the present study, we observed three behaviors, eye-tracking, and EEG findings about empathy-induced responses. First, cartoon drawing style did not have a significant effect on the three representative factors of empathy (identification, perspective-taking, and affective-sharing) and overall IES, whereas general empathy score was closely related with IES. Second, IES and subsequent attentional processing (total fixation length for gaze duration in the empathic ending scene AOI) were highly correlated when both the iconic and realistic cartoons were viewed, though no significant differences of initial attention orienting features (time to first fixation, first fixation length) were observed between the two groups. Third, iconic drawing styles suppressed gamma activity in the fronto-central area more strongly than realistic drawing.

The major finding was that there were no significant differences in any of the subfactors of IES and parameters of eye movements between the two stimuli, and this indicates that the bottom-up process (drawing style) had no considerable impact on empathy induction in our cartoons. If one drawing style was more influential, some factor would have showed more of either dominance or suppression than the other drawing style, because other experimental conditions and empathy induction abilities (GES subfactors) of the two groups were at the same level. This means that the top-down process (story plot) occurred identically in both groups regardless of the type of character drawing style. In other words, top-down process was more important than the bottom-up process in empathy induction when viewing cartoons, for the reason that empathy is not a purely sensory-driven process (Singer & Lamm, 2009). There are many studies showing that empathy is modulated more by top-down processes, such as the contextual appraisal of a situation and perspective-taking of a character rather than the sensory input variations ofbottom-up processes (Decety & Lamm, 2006; Hein & Singer, 2010; Lamm, Batson & Decety, 2007; Lamm et al., 2008). To support this viewpoint, there are some popular cartoons and art works in which the drawing style are neither fancy nor delicate (realistic) but are already being shown in online media. Most of them have unique drawing styles and solid story plots that convey exciting and empathic messages.

The presented behavioral result that the difference in drawing styles does not affect the subjective empathy scores of participants is substantially consistent with the result of a past fMRI study showing that the two different styles (pictures and cartoons) in the same pain-induced contents show no difference in subjective perception of pain (Gu & Han, 2007). Gu and Han observed a greater activation of the ACC for pictures than for cartoons and a proportional relationship between pain intensity rating (behavioral response) and the degree of activations of the ACC/paracingulate and the right middle frontal gyrus. Despite these differences in neural responses, participants reported the same ratings for the pain of others in both the pictorial and cartoon style stimuli. They concluded that paying more attention to the visual details (the lack of visual features) in cartoons made difference neural activations and empathy which were modulated by top-down controlled mechanisms (the prior knowledge of stimulus reality).

Moreover, in one previous study regarding the neural mechanisms underlying empathic pain conveyed through physical attractiveness and sex, the authors also reported no significant differences in the pain ratings of behavioral responses, despite having observed greater ACC activation for attractive women than for attractive men (Jankowiak-Siuda et al., 2015). In another psychiatry research of multi-level comparisons in patients with schizophrenia on empathy induction for cartoon tasks, the patient group showed similar behavioral responses as the healthy group (Lee et al., 2010). There were differences in activation of the fronto-temporal cortical network in each subcomponent of empathy. Nevertheless, the Interpersonal Reactivity Index test score for measuring empathic ability, response accuracy and time were not significantly different. On the other hand, Suzuki and colleagues reported that both pain intensity ratings and self-unpleasantness ratings (measured on questionnaires for behavioral experiments) were larger with human-hand stimuli than with robot-hand stimuli, although they showed an identical descending phase of the P3 component and that the degree of falling was larger for the painful stimuli than the non-painful stimuli (Suzuki et al., 2015).

Similar to the finding mentioned above, one past eye-tracking study on gazing behaviors have reported that content-related top-down processes prevail over low-level visually-driven bottom-up processes when appreciating representational paintings (Massaro et al., 2012). However, our eye movement results did not reflect top-down process behaviors (content related gazing pattern) explicitly. Because we used a single story plot and character composition in each of the four stimuli in this study, there were no comparable groups for the top-down gazing pattern. We have, however, confirmed with concrete evidence that there were no significant differences of bottom-up gazing patterns, which were the initial attention orienting and subsequent attentional processing between the two drawing styles.

Initial attention orienting may reflect pre-attentive processing of stimuli accompanied by differential implicit emotion or behavioral experience (Robinson, 1998). Pre-attentive processing is known to facilitate emotional detection and reaction (i.e., fear, threat) when stimuli are relevant to one’s existing cognitive-affective schema (Weierich, Treat & Hollingworth, 2008). Based on this theory, Tine and colleagues have revealed that participants reporting low pain intensity directed their attention more quickly to faces depicting pain (time to first fixation was short, but first fixation length was similar) than to faces depicting neutrality (Vervoort et al., 2013). Unlike this case, our stimulus did not directly recall the individual’s traumatic experiences or feelings, nor did they make the participants feel threatened or endangered. Simultaneously presenting the empathic and non-empathic scenes may encourage participants to gaze at them equally without prejudice or preference, and thus comprehend the two situations in parallel, which may result in similar time taken to the first fixation (time to first fixation and first fixation length) for the two scenes.

Regarding the total fixation length for subsequent attentional processing, both groups revealed that total fixation lengths were highly positively correlated with IES. This agrees with the findings from other studies showing that emotional tasks required more gazing time to judge aesthetic evaluations or be empathized with specific feelings implicitly (Cowan, Vanman & Nielsen, 2014; Massaro et al., 2012; Matsumoto et al., 2015; Savazzi et al., 2014; Villani et al., 2015). Moreover, the gaze duration of the not empathized scene was significantly shorter than the gazing time of non-AOIs. That is, participants usually gazed at blank spaces or outside the monitor rather than gazing at the non-empathy-induced scene for the total 7 s of the third ending scene.

Interestingly, another result indicated that each drawing style activated different brain areas during EEG measurement. Gamma band activity in the fronto-central area was associated more with the iconic drawing style than the realistic drawing style. This was apparent during the period of 1,000 ms to 1,600 ms after viewing the third ending scene. The fronto-central area is involved in the integration of sensory information and retrieved memories, which are highly correlated with emotional brain functions (Bernhardt & Singer, 2012). As mentioned in other fMRI and MEG studies in the past, the ACC, AI, IFG, dorsolateral prefrontal cortex, medial prefrontal cortex and amygdala are brain regions representative for inducing empathy, and are located at the front and middle part of the brain (Bernhardt & Singer, 2012; Decety, Michalska & Akitsuki, 2008; Fan et al., 2011; Gu & Han, 2007; Jankowiak-Siuda et al., 2015). The higher magnitude of the iconic drawing style than the realistic style may suggest that additional cognitive processes are required to understand the abstract meaning conveyed by iconic drawing styles. In this regard, Chatterjee & Vartanian (2016) suggested that distinct visual processing aspects of each drawing style were mapped onto specific brain parts. Another recent EEG and MEG study reported that gamma oscillations reflect the maintenance of feature-specific information in visual working memory while contributing to feature binding in the formation of memory representations (Honkanen et al., 2014).

Furthermore, several studies have reported that emotional functions, including empathy for negative or unpleasant expressions (i.e., sadness, depression, worry, anxiety, phobia), are correlated with gamma band activity (Güntekin & Başar, 2014; Herrmann, Fründ & Lenz, 2010; Li et al., 2015; Müller, Gruber & Keil, 2000; Oathes et al., 2008). Notably, negative pictures (angry and fearful) increased gamma oscillatory responses in comparison to neutral and/or positive pictures (Martini et al., 2012; Sato et al., 2011). Three of the four of our scenarios were classified as empathizing for negative situations (struggling) and the empathic tasks basically made subjects think about the unpleasant consequence that the opponent would face when being refused. Accordingly, our results revealed an appropriate gamma oscillation, which agrees with the previous studies.

The delayed (approximately 1 s or 1,000 ms) but high activation of frontal gamma powers in response to the iconic drawing style may be an indicator reflecting a top-down process once again. Hajcak and colleagues explained this delayed timing as the late positive potential (LPP), which represents the top-down emotion regulation and appears typically between 300 ms and 800 ms after stimulus onset (Hajcak, MacNamara & Olvet, 2010). The LPP is a midline ERP that becomes evident approximately 300 ms following stimulus onset. If the gamma activation appeared much later than 2,000 ms, this modulation would reflect a closed-loop system, including feedback from the appraisal system, which is associated with bottom-up emotional regulation (Chiesa, Serretti & Jakobsen, 2013).

Obviously, drawing in the realistic style requires more time and effort by the cartoon artist. However, our research shows that it may be more efficient to focus on fast storytelling when creating cartoon content when time and resources are limited. This might be the best way for us to take full advantage of the characteristics of the cartoon media. It is also a great advantage for the One Source Multi Use (OSMU) demand, which has been spotlighted in the content market these days. Cartoons are superior to text-based novels in conveying story, feeling and emotion, but they cost much less to produce than movies, dramas, games, plays and other media. For this reason, cartoons could be a good pilot test before starting large-scale content making business. From a new media perspective, neurophysiological measures could be used to enhance the viewing experience in accordance with personal preferences (Johannisson, 2016; Maskeliunas & Raudonis, 2016) and adjust the customized story plot in making interactive E-books (Li et al., 2016; Maskeliunas et al., 2016; Van Erp, Hogervorst & Van der Werf, 2016).

Although our results clarify the modulation of empathy via information processing of cartoons, our study had some limitations that will be addressed in the future studies. First, we did not entirely rule out the influences of other emotional or motivational factors, such as preference, interest, gender, and background experience because of the small number of participants. Therefore, future studies may also need to perform comparative analysis on such factors after changing the stimulus and procedure that includes non-empathy induction choice evidently. Second, we did not consider empathy for complex emotions in this study. Stories conveyed by cartoons are typically much longer and contain various types of emotions for each situation and character concurrently. Therefore, further studies should analyze the detailed area of interest for each scene, which is largely divided into the face and body actions (including hand information) of the character (Tao & Sun, 2013).

## Conclusions

We investrigated the possibility of empathy induction during cartoon viewing using eye-tracking and EEG. By changing the drawing style of the cartoon characters, we confirmed the advantages of the abstraction of cartoons and found a relationship between bottom-up processes and top-down processes that occur while watching cartoons. This was because cartoons in iconic drawing style induced levels of empathy similar to the levels induced by cartoons in realistic drawing style, even though the iconic drawing style depicted highly abstract story plots in a less detailed manner. Though the two drawing styles caused activations of the gamma band in different brain regions, this difference did not affect the degree of inducing empathy. Interestingly, gaze duration of cartoons was proportional to IES.

##  Supplemental Information

10.7717/peerj.3988/supp-1 Data S1 Behavioral measures (General Empathy Scale and Induced Empathy Score) and eye-tracking measures

## Additional Information and Declarations

### Data Availability

The following information was supplied regarding data availability: The raw data has been provided as a Supplemental File.
